# Supplementary material for: Phenotypic and Genotypic Virulence Characterisation of Staphylococcus pettenkoferi Strains Isolated from Human Bloodstream and Diabetic Foot Infections
Source: Int J Mol Sci. 2022 Dec 7;23(24):15476. doi: 10.3390/ijms232415476 (PMC9778964; doi:10.3390/ijms232415476)
Supplement: Supplementary file 1 [file ijms-23-15476-s001.zip › ijms-2021840-figures.pdf]

Supplementary figures

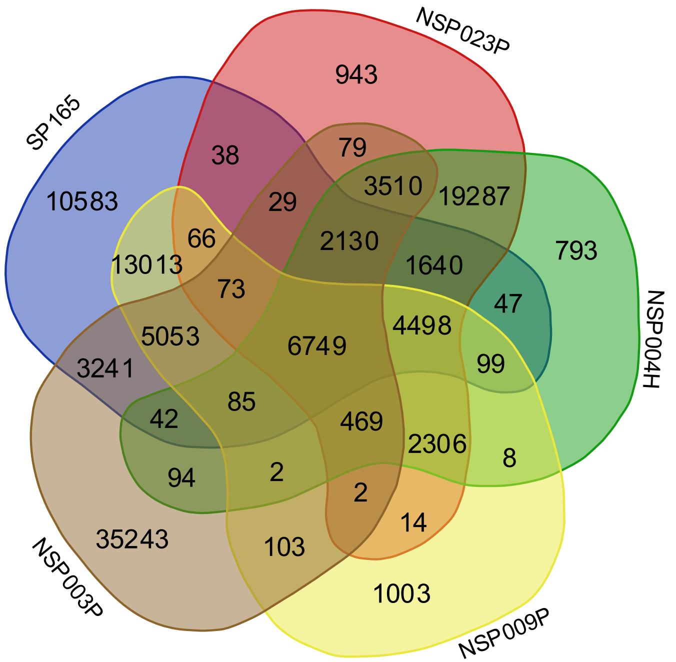

Figure S1: Single nucleotide polymorphism (SNP) analysis of *S. pettenkoferi* SP165, NSP003P, NSP009P, NSP023P and NSPH004H using the genome reference FDAARGOS_288 (GenBank accession n° NZ_CP022096). The diagram was constructed with Venn diagram (Draw Venn Diagram (ugent.be) based on SNP difference investigation using snipy on galaxy online platform (https://www.usegalaxy.org.au/).

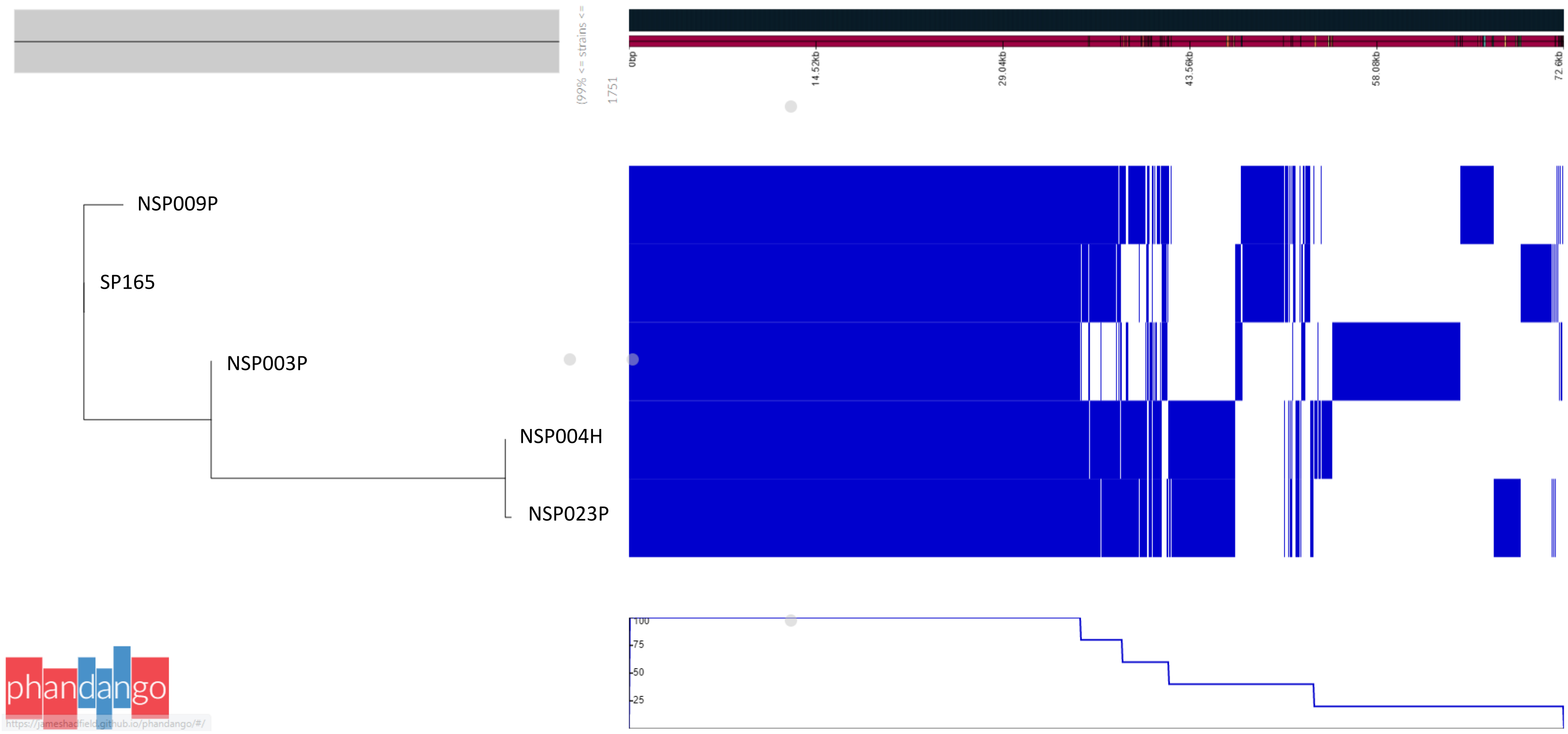

Figure S2: Pangenome analysis of *S. pettenkoferi* SP165, NSP003P, NSP009P, NSP023P and NSPH004H strains. NSP009P clustered with SP165 that explaining the high virulence level expressed by both strains. The same clustering was observed with strains NSP004H and NSP023P with low virulence level. The clustering distance of the strain SNP003P could be confirmed by the genomic distinction previously documented by whole genome annotation.
