# Supplementary material for: Phenotypic and Genotypic Virulence Characterisation of Staphylococcus pettenkoferi Strains Isolated from Human Bloodstream and Diabetic Foot Infections
Source: Int J Mol Sci. 2022 Dec 7;23(24):15476. doi: 10.3390/ijms232415476 (PMC9778964; doi:10.3390/ijms232415476)
Supplement: Supplementary file 1 [file ijms-23-15476-s001.zip › Supplementary Materials-caption.pdf]

Table S1: Summary of the whole-genome sequencing bioinformatic analysis of *S. pettenkoferi* strains; Table S2: Resistome traits of *S. pettenkoferi* strains; Table S3: Plasmid research in the genomes of *S. pettenkoferi* strains; Table S4: Growth profiles of *S. pettenkoferi* NSP003P, NSP009P, NSP023P and NSP004H and *S. aureus* Newman; Table S5: Genomic analysis of biofilm-associated genes in *S. pettenkoferi* strains; Table S6: *S. pettenkoferi* whole-genome-based analysis to determine putative virulence determinants; Figure S1: Single-nucleotide polymorphism (SNP) analysis of *S. pettenkoferi* SP165, NSP003P, NSP009P, NSP023P and NSPH004H using the genome reference FDAARGOS_288 (GenBank accession n° NZ_CP022096). The diagram was constructed with Venn diagram (Draw Venn Diagram (ugent.be)) based on SNP difference investigation using snipy on the galaxy online platform (https://www.usegalaxy.org.au/ (accessed on 27 November 2022)). Figure S2: Pangenome analysis of *S. pettenkoferi* SP165, NSP003P, NSP009P, NSP023P and NSPH004H strains.
